# Flexible and Accurate Detection of Genomic Copy-Number Changes from aCGH

**DOI:** 10.1371/journal.pcbi.0030122

**Published:** 2007-06-22

**Authors:** Oscar M Rueda, Ramón Díaz-Uriarte

**Affiliations:** Structural and Computational Biology Programme, Spanish National Cancer Centre (CNIO), Madrid, Spain; Lilly Singapore Centre for Drug Discovery, Singapore

## Abstract

Genomic DNA copy-number alterations (CNAs) are associated with complex diseases, including cancer: CNAs are indeed related to tumoral grade, metastasis, and patient survival. CNAs discovered from array-based comparative genomic hybridization (aCGH) data have been instrumental in identifying disease-related genes and potential therapeutic targets. To be immediately useful in both clinical and basic research scenarios, aCGH data analysis requires accurate methods that do not impose unrealistic biological assumptions and that provide direct answers to the key question, “What is the probability that this gene/region has CNAs?” Current approaches fail, however, to meet these requirements. Here, we introduce reversible jump aCGH (RJaCGH), a new method for identifying CNAs from aCGH; we use a nonhomogeneous hidden Markov model fitted via reversible jump Markov chain Monte Carlo; and we incorporate model uncertainty through Bayesian model averaging. RJaCGH provides an estimate of the probability that a gene/region has CNAs while incorporating interprobe distance and the capability to analyze data on a chromosome or genome-wide basis. RJaCGH outperforms alternative methods, and the performance difference is even larger with noisy data and highly variable interprobe distance, both commonly found features in aCGH data. Furthermore, our probabilistic method allows us to identify minimal common regions of CNAs among samples and can be extended to incorporate expression data. In summary, we provide a rigorous statistical framework for locating genes and chromosomal regions with CNAs with potential applications to cancer and other complex human diseases.

## Author Summary

As a consequence of problems during cell division, the number of copies of a gene in a chromosome can either increase or decrease. These copy-number alterations (CNAs) can play a crucial role in the emergence of complex multigenic diseases. For example, in cancer, amplification of oncogenes can drive tumor activation, and CNAs are associated with metastasis development and patient survival. Studies on the relationship between CNAs and disease have been recently fueled by the widespread use of array-based comparative genomic hybridization (aCGH), a technique with much finer resolution than previous experimental approaches. Detection of CNAs from these data depends on methods of analysis that do not impose biologically unrealistic assumptions and that provide direct answers to fundamental research questions. We have developed a statistical method, using a Bayesian approach, that returns estimates of the probabilities of CNAs from aCGH data, the most direct and valuable answer to the key biological question: “What is the probability that this gene/region has an altered copy number?” The output of the method can therefore be immediately used in different settings from clinical to basic research scenarios, and is applicable over a wide variety of aCGH technologies.

## Introduction

Alterations in the number of copies (gains, losses) of genomic DNA have been associated with several hereditary anomalies and are involved in human cancers [1–7]. For example, amplification of some genes, especially oncogenes, is one well-known mechanism for tumor activation [8,9], and it is involved in the deregulation of cellular control [10,11]. Copy-number alterations (CNAs) have been associated with tumoral grade, metastasis development, and patient survival [1–7], and studies about copy-number changes have been instrumental for identifying relevant genes for cancer development and patient classification [1,2,12].

A widely used technique to identify copy-number changes in genomic DNA is array-based comparative genomic hybridization (aCGH). Two DNA samples (e.g., problem and control) are differentially labeled (often with fluorescent dyes) and competitively hybridized to chromosomal DNA targets. After hybridization, emission from each of the two fluorescent dyes is measured, and the signal intensity ratios are indicative of the relative copy number of the two samples [1,2,13]. Therefore, a key step in any study of the relationship between altered copy numbers and disease is using the fluorescence ratio data to identify genes and contiguous chromosomal regions with altered copy numbers.

The main biomedical problem, both for the study of the CNAs per se and for downstream analysis (e.g., relationship with gene expression changes or patient classification), is the accurate identification of the genes/chromosomal regions that have an altered copy number. Satisfactorily dealing with this problem requires a method that (1) provides direct answers that can be used in different settings (e.g., clinical versus basic research), (2) reflects the underlying biology and accounts for key features of the technological platform, and (3) can accommodate the different levels of analysis (types of questions) addressed with these data.

First, estimates of the probabilities of alteration (instead of *p*-values or smoothed means) are the most direct and usable answer to this problem [14,15]. Probabilities can be used in contexts that cover basic research to clinical applications [1,2] so that, for instance, a clinician might require high certainty of alteration of a specific gene before more invasive procedures, whereas a basic researcher can consider for further study genes that show only a moderate probability of alteration (e.g., probability >0.5). Finally, appropriately used, probabilities of alteration can account for uncertainty in model building [16,17].

Second, the analysis should incorporate distance between probes [2,15,18–21]: widely used aCGH platforms such as those based on cDNA microarrays, oligonucleotide arrays, and representational oligeonucleotide microarray analysis (ROMA) lead to variable coverage across chromosomes, with unequal distances between probes (i.e., some regions have probes that are very close to each other, whereas in other regions probes are very far apart). As copy-number changes involve chromosome segments, contiguous loci will have the same copy number, unless there is an abrupt change to another copy number [1,22]: the farther apart two loci are, the more likely it is that a copy-number event will have taken place in between them. Thus, in densely covered regions, the copy number of a probe is a good predictor of the copy number of the neighboring probes. In contrast, in poorly covered regions, contiguous probes or loci might be many thousands of kilobases apart, making it more likely that at least one copy-number change has taken place, and consequently, a probe provides less information about the likely state of its neighboring probes. Therefore, unless we use a platform where all probes are equally spaced, we need to use the distance between probes (and not just the order) so that the information that consecutive probes provide is adequately accounted for.

Third, depending on the focus of the study, the analysis should be conducted either chromosome by chromosome, or genome-wide [14–16]. Analyses at the chromosome level are appropriate to detect alterations in copy numbers of loci relative to the rest of the loci in that same chromosome, regardless of that chromosome's ploidy (a trivial example would be detection of copy-number changes in loci of the human Y chromosome in an otherwise diploid genome). On the other hand, detection of copy-number changes that affect most of a chromosome often require genome-wide analysis (in chromosome-wide analysis, as the mean or median chromosome level is used as the reference, detection of such changes is virtually impossible). Moreover, the use of genome-wide analysis can offer statistical advantages (e.g., reduced variance of estimation). As both types of analysis offer complementary information because they focus on different biological phenomena (chromosomal gains/losses versus gains of loci within chromosomes), a suitable method should allow these two approaches.

### Previous Approaches

Available methods for the analysis of aCGH fail some or most of these requirements. Smoothing techniques [21,23–28] do not use interprobe distance, nor do they provide posterior estimates of the likely state of each probe/clone, and data from each chromosome are analyzed independently of each other. Hidden Markov models (HMMs) and related techniques offer a flexible modeling framework, and can provide probabilities of alteration [14–16]. Some HMM-based methods [16,19], however, do not incorporate the distance between probes, assuming instead that interprobe distance is constant. In addition, most of them do not deal satisfactorily with the unknown number of hidden states (the true number of states of copy number). Some methods fix in advance the number of hidden states to three [14,15] or four [16]: prespecification of the number of states has the consequence of jumbling all changes involving multiple gains into a single state with a common mean, which is biologically questionable [22], especially as the resolution of the technology improves. Moreover, the identification of important genes for disease sometimes requires examining the amplitude of CNAs and not just their presence and location [1]; collapsing states into three or four, however, precludes examining in fine enough detail the amplitude of CNAs. A better approach would provide posterior probabilities of the number of states; using such a procedure over many different experiments will tell us whether three- or four-state models are a reasonable simplification. Of those methods that do not assume a fixed number of hidden states [18,19,22], one of them [22] cannot be used for questions about the number of hidden states, or for breaking the data into more categories than gained/lost/no change, which are increasingly important questions with higher-resolution techniques and are needed for distinguishing regions of moderate copy gains from regions of large copy gains; see also above for relationship between amplitude of CNAs and presence of disease genes. The remaining two [18,19] fit HMMs for a range of number of states and then use Akaike information criterion (AIC)–based model selection, but AIC-based selection with HMMs has not been theoretically justified [29] and does not provide a probability of the likely number of states; moreover, selecting a single model leads to underestimation of the true variability in the data. These two methods, in addition, use a final clustering step of hidden states that introduces several ad hoc decisions.

### Statistical Model: Overview

We have developed a method, reversible jump aCGH (RJaCGH), that fulfills the three requirements above, and does not suffer from the limitations discussed for other methods. Our method is applicable to aCGH from platforms including ROMA, oligonucleotide aCGH (oaCGH; including Agilent, NimbleGen, and many noncommercial, in-house oligonucleotide arrays), bacterial artificial chromosome (BAC), and cDNA arrays [1,13]. We start our modeling by noting that, for a given chromosome or genome, the copy numbers of genomic DNA (e.g., 0, 1, 2 copies, . . . ) of different probes or segments are an unknown finite number. Thus, probes or segments could be classified into several groups with respect to their (unknown) copy number. In addition, as mentioned above, we expect that the copy number of a probe will be similar to the copy number of its closest neighbors, with that expected similarity decreasing when probes are farther apart. Finally, for a given copy number, the aCGH fluorescence ratios should be centered around a log_2_ value, with some random noise. We want to use the observed log-ratios to identify regions with altered copy number.

The biological features of this model (a finite number of unknown or hidden states that are indirectly measured, with states of close elements likely to be similar, and variable distances between probes) can be modeled with a nonhomogeneous HMM [29]. To provide a direct estimate of the probability that a given probe or region has an altered copy number, we use a Bayesian model computed via Markov chain Monte Carlo (MCMC). Since we do not know the true number of hidden states, we fit models with varying numbers of hidden states and, to allow for transdimensional moves between models with different numbers of states, we used reversible jump [30]. After running a large number of MCMC iterations, we can summarize the posterior probabilities. First, we obtain posterior probabilities for the number of states. Conditional on a given number of states, each model provides posterior distributions of the parameters of interest (e.g., means, variances, transition matrices). From the latter, we can obtain posterior probabilities that a probe is gained or lost. To obtain our final estimates, we incorporate the uncertainty in model selection by using Bayesian model averaging [17], with estimates weighted by the posterior probability of each number of states, for the probabilities of probes being gained or lost. We call the complete statistical method RJaCGH (from reversible jump–based analysis of aCGH data).

## Results

We applied RJaCGH and the best performing alternative methods (based on two recent reviews [20,31]) to the 500 simulated datasets of [31] (see also Protocol S1). These are data “...simulated to emulate the complexity of real tumor profiles” and designed to become “...a standard for systematic comparisons of computational segmentation approaches,” [31] and are not data simulated under our own model. To assess the effect of variable interprobe distance, we randomly deleted data points (see details in Protocol S1) so that each original simulated dataset gives rise to another four datasets with (an average of) 10%, 25%, 50%, and 65% of observations missing. The length of these gaps is modeled by a Poisson distribution, so larger percentages of missing data correspond to larger variability in interprobe distances.

**Figure 1:**
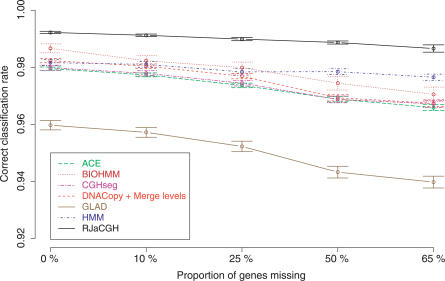
Effects of Variability in Interprobe Distance (Percentage of Probes Missing) on Correct Classification Shown are the mean and 95% confidence interval around the mean of the correct classification error rate. Each mean and confidence interval is computed from 500 datasets [31]; see text and Protocol S1 for generation of interprobe distance variability. Alternative methods compared are ACE, developed by [27]; BioHMM, a nonhomogeneous HMM by [18]; DNAcopy with mergeLevels, with the original method developed by [24] (and use of mergeLevels following [31]); HMM, a homogeneous HMM developed by [19]; CGHseg, a random Gaussian process with abrupt changes in the mean by [28]; and GLAD, which uses a nonparametric likelihood method with adaptive weights for breakpoint detection, by [23].

**Figure 2:**
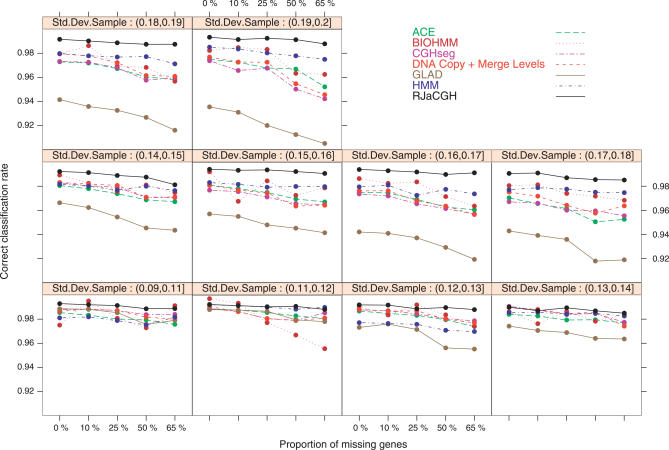
Joint Effects of Variability in Interprobe Distance and Noise on Correct Classification The same data are shown as those in Figure 1. The noise (standard deviation) of each sample is split into ten non-overlapping ranges, and each panel shows the mean correct classification success versus the proportion of missing probes (i.e., increasing levels of variance in interprobe distance). Each mean is based on approximately 50 samples. See Figure 1 for method references.

Results in Figure 1 (see also Figure 1 in Protocol S1) show the excellent performance of RJaCGH, and how it outperforms alternative methods. Moreover, Figure 2 (see also Figures 2 and 3 in Protocol S1) shows that the difference between RJaCGH and alternative approaches is accentuated when we consider jointly the effects of noise and variability in interprobe distance. Analysis using three other performance statistics (false discovery rate, sensitivity, and specificity) show the same overall patterns (see Protocol S1, Figures 2 and 3): for some specific statistics, RJaCGH can be second (but very close) to another approach; this other approach, however, performs poorly with respect to the remaining statistics.

This paper focuses on the statistical performance of the methods compared. In terms of speed, nevertheless, our approach is clearly the slowest one. We are currently working on improving the speed of the execution both by using more efficient algorithms and by using parallel computing.

Similar results are obtained when applying these methods to a real dataset of nine cell lines [32], and when comparing the predicted ploidy with the known ploidy (see Protocol S1, Figure 4). Overall, therefore, there is strong evidence that RJaCGH is the best performing of the existing methods.

## Discussion

The excellent performance of RJaCGH is a result of the statistical method used, which is essentially a careful and rigorous development from first principles. We set out to obtain a method that allows us to seamlessly incorporate interprobe distances (to allow usage over varied technological platforms), that makes no untenable assumptions about the true number of copy levels (since this is likely to vary between datasets), that permits analysis at the chromosome and the genome level, and, finally, that returns posterior probabilities of alteration, because these posterior probabilities constitute the direct answer to the basic biomedical question (“Is this gene likely to have an altered copy number?”).

Based simply on our usage of interprobe distance, we should expect RJaCGH to perform better than all alternative approaches, with the possible exception of BioHMM [18], as interprobe distance variability increases. Moreover, RJaCGH adapts to variable noise in the data, without the need for fine-tuning of parameters (all results reported are obtained from the default settings of RJaCGH). As noted above, the relative advantage of RJaCGH increases as the interprobe variability increases and the noise in the data increases, which shows that our theoretical developments have practical consequences and emphasizes the importance of both accounting for interprobe distance and appropriately modeling variance in the data.

In addition, we use Bayesian model averaging, which has been repeatedly shown [33] not only to account for uncertainty in model selection but also to lead to point estimators and predictions that minimize mean square error. On its own, our usage of Bayesian model averaging could be largely responsible for the better performance of RJaCGH over all other methods, even in the absence of interprobe distance variability and when there is low noise in the data (left of Figure 1, and left of bottom-row panels in Figure 2). In addition, reversible jump allows us to consider a variety of models (regarding number of states), and its birth and split moves are also beneficial for a more thorough exploration of the posterior probability (within a model with a given number of states) when the density is multimodal. Finally, our method, in contrast to other approaches (e.g., DNAcopy), can identify single-clone aberrations, which might be key for large-scale genomic deregulation if the single-clone aberrations affect certain specific genes or promoters; for example, the inability to detect single-gene alterations is shown to have an effect in a study of pancreatic adenocarcinoma [5], where the loss of the *SMAD4* tumor suppressor is undetected.

In addition to features that can be compared with other methods, RJaCGH has two unique features that set it apart from most alternative approaches. First, the user can analyze data at either the genome or the chromosome level, thus addressing different types of questions. Some approaches (e.g., BioHMM, HMM, GLAD, DNAcopy) allow us to perform genome-wide inferences, but they use essentially an ad hoc postprocessing of results of analysis that is conducted at the chromosome level. Finally, one of the main features of RJaCGH, its returning of posterior probabilities of CNAs, simply cannot be compared with most alternative methods as they do not provide this type of output. What most alternative approaches return are smoothed means, *p*-values, or a classification into states without any assessment of the uncertainty of this assignment to states. But a probability of alteration (which RJaCGH returns) is much easier to interpret and to use (with possibly different thresholds depending on the type of research question), and is often the direct answer to the basic biomedical question. The few alternative approaches that return probabilities of alteration [14–16] all make the untenable assumption that the true number of biological states of alteration is three [14,15] or four [16].

Directly returning probabilities of alterations has profound consequences, both for current practices and for future developments. As argued above, these probabilities are the direct answer to the question “Does this gene have an altered copy number?”; *p*-values or smoothed means are not a direct (and often not even an indirect) answer to that question. In addition, the improvement in the resolution of aCGH techniques [2,13] is increasingly allowing for multiple assayed spots per gene. Probabilities of alteration for each spot can be combined to find the gene-level probability of alteration, a distinct advantage over smoothed means or *p*-values.

For currently active research areas, the availability of rigorously obtained probabilities of alterations has far-reaching consequences, both in terms of the biological phenomena that can be exposed and as an avenue of further research. First, the availability of probabilities of alteration should improve the identification of regions with consistent alterations across samples [34,35] in a statistically rigorous way (including, if needed, control of false discovery rate), and the detection of subgroups of samples according to recurrence patterns [4,35,36]. Critical disease genes are often located in CNAs that are recurrent across individuals and that have at least some high-amplitude changes [1,35,37], and analysis of aCGH data has allowed us to identify subgroups, within established diseases, that could have therapeutic relevance (e.g., in glioblastoma [4]). Available methods use the assignment of each gene to one state (equivalent to assuming that there is complete certainty in this assignment); however, we would not want to give the same weight, when looking for minimal common regions, to a gene with a probability of being gained of 51% and to a gene with a probability of being gained of 90%, since this practice will lead to a coarser definition of boundaries and can even preclude the detection of some minimal regions altogether. The inherent limitations of methods that use a simple categorization into gain/loss/no change with an assumed 100% certainty have already been recognized by some of the developers of such methods [35]. Moreover, incorporation of amplitude of change, which might be a crucial feature of minimal common regions that harbor critical disease genes [1,5,35,37], is not feasible with some methods [34], but should be straightforward by combining posterior probabilities and posterior means of each state, as returned from RJaCGH.

Second, posterior probabilities of being in a specific state, together with the estimated posterior mean of each state, can be used as the basis for a statistically rigorous and biologically sound approach for identifying breakpoints. At present, the identification of breakpoints depends completely on the resolution of the method, and does not allow us to combine the probability of membership in different states with the biological relevance of an estimated mean difference; however, the precise definition of boundaries and amplification maxima are important not only for the study of genomic copy numbers, but also for understanding the relationship between aCGH and expression data [38].

Third, the model of RJaCGH can be extended to provide rigorous downstream analysis of aCGH, including patient classification [1,31] and the integration of gene expression and proteomic data [12,31]. CNA data analysis, compared with mRNA expression data, can be performed on formalin-fixed paraffin-embedded material, and CNAs define key events that drive tumorigenesis, and thus are probably more valuable as prognostic markers and as predictors of treatment response [39,40]. Improved resolution of CNA data analysis, however, can be crucial in obtaining very valuable classifiers, as evidenced from the “almost success” of some studies attempting to differentiate *BRCA2* from *BRCA1, BRCAX,* and sporadic cases in breast tumors (see discussion in [40]); the finer resolution provided by probabilities and posterior mean estimates might be pivotal here. Incorporating expression and proteomic data, on the other hand, is the basis for the identification of copy-number changes that are significant in the development of disease [1,41,42]. Since changes in copy number are not always reflected in changes in expression [1,5], analytical methods that provide finer resolution are crucial. Moreover, within a probabilistic framework it is possible to systematically and rigorously address questions of how CNAs in a given chromosome affect expression changes in genes located in other chromosomes, an increasingly important research question [43]. Finally, the posterior probabilities and means returned from aCGH can be considered as denoised [44,45] signals from the log_2_ aCGH ratios that reflect underlying copy number variation; as such, these are highly relevant to the recent studies on the relationship between copy-number variation and complex phenotypes [46,47], which emphasize the importance of copy-number variation in genetic diversity and disease in humans.

## Materials and Methods

### Model.

We use a nonhomogeneous HMM with Gaussian emissions. We can either fit one model to all the chromosomes of an array, or we can fit a different model for each chromosome of an array. Let *n* be the number of probes, and *k* the number of different copy numbers in the collection of probes. Let *S_i_* be the true state (copy number) of the probe *i*: *S_i_* = {*1, …, k*}*_i_*_=1,...,*n*_. Let *Y_i_* be the relative copy number of the probe *i*, that is the log ratio of fluorescence intensities between tumor and control samples. Let *d_i_* be the distance in bases between probe *i* and probe *i +* 1. How distance is measured depends on the platform: distance can be the distance from the end of the spot to the start of the next, if the length of the spots is proportional to the length of the probe (so we have the same information for every probe), or the distance between the midpoint of the spots, if the length of the spots is not proportional to the length of the probe. We normalize these distances between 0 and 1 to increase numerical stability (with probes in adjacent bases with a scaled distance of 0).

We assume that {*S_i_*} follows a nonhomogeneous first-order Markov process, as: *P*(*S_i_* = *s_i_* | *S_i_*_−1_ = *s_i_*_−1_, *X_i_*_−1_ = *x_i_*_−1_) = 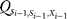 Biologically, we expect that 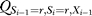 ,the probability of staying in the same hidden state, is a decreasing function of *X_i_*_−1_, so the dependence of the state of a probe onto the next one is lower the farther the probes are. We also expect that when the distance between two probes is maximal, the state of a probe should be independent from the state of its predecessor. Thus, we model the transition probabilities as:

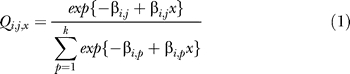
where *β* has the form:

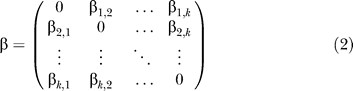
with all *β_ij_* ≥ 0 *∀ i, j*. Finally, conditioned on {*S_i_*}, {*Y_i_*} follows a Gaussian process: 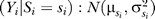


Similar approaches have been used before with nonhomogeneous HMM [48,49]. In our case, the transition matrix should fulfill the following biologically based properties: (1) the probability of remaining in the same hidden state should be a decreasing function of the distance between a probe and the previous probe; and (2) when the distance between two probes is maximal, the state of a probe should not be affected by the state of the previous probe. With the above parameterization, and since the diagonal of *β* is zero (which is also needed for the parameters to be uniquely defined), the probability of remaining in the same state *i* is 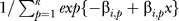 , a decreasing function of distance (*x*). Moreover, as distances are scaled between 0 and 1, when the distance between two probes is 1, the probability of staying in the same state is *1 / k*, where *k* is the number of states; therefore, when the distance is maximal, the state of a probe does not depend on the state of the previous probe. (The value of this “maximal distance” beyond which two probes are considered independent is a parameter to the model, and can be adjusted taking into account the specific array characteristics).

For computational reasons and modeling flexibility, we opted for Bayesian methods using MCMC. To fit models with varying number of hidden states, we used reversible jump. Suppose that we have a collection of *K* HMM models, and each of them has a number of *k* hidden states, from *k =* {*1, . . ., K*}. Let *θ*(*k*) be the HMM associated to *k*, that is, *θ*(*k*) *=* {*μ*(*k*)*, σ* ^2^(*k*)*, β*(*k*)}. The prior distributions for the model are the usual ones in mixture problems [50]: *p*(*k*) is the prior for the number of hidden states with *p*(*k*) *~ U*(*1,k*), *p*(*θ*(*k*)| *k*) is the prior of the HMM conditioned to *k*, the number of hidden states with *u*(*k*) ~ *N*(*α*,*ϱ* ^2^), where *α* and ϱ are the median and range of *Y_i_*; *σ* ^2^(*k*) *~ IG*(*ka, g*), where *ka* is 2 and *g* is *ϱ* ^2^(*Y_i_*) / 50; *βk*) ~ *Γ*(*1, 1*). The likelihood of the model, *L*(*y; k, θ*(*k*)) can be computed by forward filtering [29], so the joint distribution is *p*(*k*)*p*(*θ*(*k*)|*k*) *L*(*y; k, θ*(*k*)).

### Estimation and fitting.

We can draw samples from the posterior distribution through a reversible jump MCMC (RJMCMC) algorithm [30]. In RJMCMC, we explore the posterior distribution of possible models, jumping not only within a model but also between models with a different number of parameters. To match the difference between degrees of freedom, some random numbers *u* with density *P*(*u*) are generated, so if we are in state *x*, the new one is proposed in a deterministic way *x′*(*x,u*). The reverse move is the inverse of that function: *x*(*x′,u′*). This way, the usual Metropolis-Hastings acceptance probability can be computed [50]:

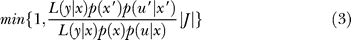
where *L*(*y* | *x)* is the likelihood, *p*(*x*) are the priors, *p*(*u | x*) are the densities of the candidates, and 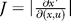 the determinant of the Jacobian of the change of variable. We combine several Metropolis steps in a sweep [29,51].

(1) Update HMM of a model using a series of Metropolis-Hastings moves. (We do not use Gibbs Sampler to avoid the hidden state sequence from becoming part of the state space of the sampler, so dimensionality is reduced and reaching convergence is easier). 

(2) Update model (birth/death). When we have *r* states, a birth/death move is chosen with probabilities *p_birth_*(*r*) and *p_death_*(*r*) (these are 1/2 except in the cases when no movement of that type can be made, [e.g., a death move when there is only one state]). If a birth move is selected, a new state is created from the prior distributions and accepted with probability

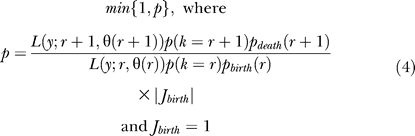
If a death move is chosen, a random state is deleted with a probability inverse to Equation 4.

(3) Update model (split/combine). A split/combine move is attempted with probabilities *p_split_*(*r*) and *p_combine_*(r) (again, 1/2 except when a move cannot be made). If a split move is selected, an existing state *i*_0_ is split into two, *i*_1_, *i*_2_:

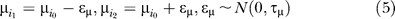


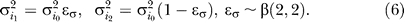
Split column

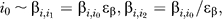


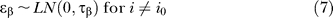
Split row

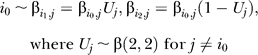


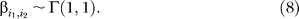
This move is accepted with probability

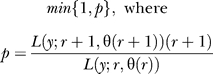


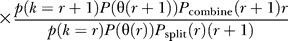


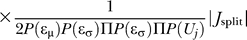


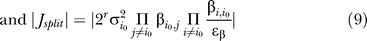
The split move must follow the adjacency condition [50]: the resulting states must be closer between them than to any other existing ones. If a combine step is selected, the symmetric move is performed, and the inverse probability of acceptance is computed.

The combination of birth and split moves makes it possible not only to visit models with a different number of parameters, but also to explore more thoroughly the posterior probability in the case of a parameter with a multimodal density.

These moves are common ones [29,51], but we have changed several aspects of their design to improve the probability of acceptance, which is the most difficult step in reversible jump [29,30,51]. We constrain the variance of every state so that it cannot be greater than the variance of the entire data. Also, we have added the adjacency condition mentioned before, and used centering proposals [52]. To prevent label-switching of states, we have ordered the states according to means after every iteration of the sweep [50].

### Inference.

We run the former algorithm a large number of times (e.g., 50,000) and, after discarding the first iterations as burn-in, we keep the last (e.g., 10,000) samples as observations from the joint distribution so that we can make inferences from it. For every model that has been visited, we obtain the posterior probabilities of the mean copy number of every state, the variance of the copy number of every state, and the function of transitions between hidden states. By counting the number of times that each model has been visited, we obtain an estimate of the posterior probability of each model (i.e., we avoid using Bayesian information criterion [BIC] or AIC). Then, applying the Viterbi algorithm [29] to every sample obtained from the MCMC, and, as this sample is a function of the HMM, we can obtain its posterior probability, something that usual Viterbi cannot. From the Viterbi paths for all the samples, we can then compute the posterior probability that a probe belongs to every state or the probability that a sequence of probes is in a given state.

When obtaining posterior probabilities of copy-number change, we use Bayesian model averaging [17] over all models visited. Let *S_i_* be the lost, gained, no-change status of probe *i*, *K* the set of the models considered (in our case, that would be HMMs with 1, . . ., *K* number of states), *M_k_* the model with *k* number of states, and *S_i_* | *M_k_* the state of probe *i* according to model *k*. We compute the unconditional (with respect to model selection) probability for the probe *i* as:

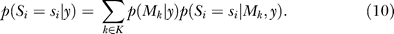



### Checking convergence and influence of priors.

As in any MCMC approach, it is crucial to assess convergence of the sampler. We follow common practice [53] of running several chains in parallel. The convergence of the sampler depends strongly on the distribution of the candidates in Metropolis-Hastings. That is, for every iteration, a new value for the parameters is proposed from a distribution centered in their current values. The standard deviation of that distribution must be chosen in a way that samples explore all the parameter space. These standard deviations are not parameters of the model in the sense that different values give different fits, but values that can speed up convergence of the algorithm. The convergence of the posterior probability of the number of hidden states is reached when a large enough number of transdimensional moves is made. This number need not to be large if the likelihood is substantially higher in a particular model and data size is big enough. The birth and death moves only depend on the priors, but the split and combine moves depend also on their own design and the values of *τ_μ_* and *τ_β_* (see Equation 5 and Equation 7). The priors chosen have been extensively tested in mixture models [50]. In addition, the priors and rest of the parameters have very little effect: even small CGH arrays contain thousands of points, so that the likelihood from the data dominates any prior. With the 2,500 simulated datasets analyzed, we have only needed to specify the number of burn-in—50,000—and to-keep samples—10,000, and the number of chains—four, and in only nine cases was there evidence of nonconvergence, which was solved by rerunning the samplers.

### Implementation and analysis.

We have implemented RJaCGH using C (for the sweep algorithm) and R [54], and all analysis and comparisons have been done in R. The code that implements RJaCGH is freely available from the usual Comprehensive R Archive Network (CRAN) repositories as package RJaCGH (http://cran.r-project.org/src/contrib/Descriptions/RJaCGH.html) or from the repository at Launchpad (https://launchpad.net/rjacgh). All data and code used for this paper are also publicly and freely available (see details in Protocol S1).

## Supporting Information

Protocol S1 Supplementary Material (551 KB PDF)

## Abbreviations

aCGH: array-based comparative genomic hybridization
CNA: copy-number alteration
HMM: hidden Markov model
MCMC: Markov chain Monte Carlo
RJaCGH: reversible jump aCGH
RJMCMC: reversible jump MCMC
