# Supplementary material for: Flexible and Accurate Detection of Genomic Copy-Number Changes from aCGH
Source: PLoS Comput Biol. 2007 Jun 22;3(6):e122. doi: 10.1371/journal.pcbi.0030122 (PMC1894821; doi:10.1371/journal.pcbi.0030122)
Supplement: Supplementary file 1 [file pcbi.0030122.sd001.pdf]

Protocol S1 for: “Flexible and accurate detection of genomic copy-number changes from aCGH”

Oscar M. Rueda and Ramón Díaz-Uriarte
Statistical Computing Team,
Structural and Computational Biology Programme,
Spanish National Cancer Centre (CNIO)
Melchor Fernández Almagro 3
28029 Madrid
Spain
E-mail: omrueda@cnio.es, rdiaz02@gmail.com

1 Method comparisons: general

1.1 Methods compared

We have examined the performance of our method and compared it two six other methods: DNA copy [1], GLAD [8], CGHseg [9], and ACE [2], approaches that have been reviewed in [3, 4], where DNAcopy and CGHseg stand as the best overall performers; we have also included in the comparisons the HMM [5] and non-homogeneous HMM [6] approaches, two methods that share some common features with our method (but see discussion). All of these approaches, except ACE, are available as R/BioConductor packages. ACE is available as a Java program from [2]; however, this Java program is not suitable for batch processing of simulations; thus, we implemented it as a loadable C module, and call it from R. Other promising methods (specially [7]) could not be included in the comparative study because code is not available or directly implementable from the available published descriptions.

1.2 Settings of methods

All methods were run with their default parameters. Details and modifications follow.

For DNA copy, and following the recommendations in [3], we have used the "merge levels" proposal of [3]. The methods of Fridlyand et al. [5] and Marioni et al. [6] include an internal, implicit, merge levels-like algorithm.

For ACE [2] the FDR used is the minimal one of the available (experimenting with the method in these data set showed that other, larger, FDRs lead to much poorer performance).

GLAD [8] was run using default parameters, and using the default approach for post-segmentation merging. Although the performance of GLAD might improve by fine tuning some of its many parameters, none of those have an intuitive interpretation, nor is there any indication as to how these parameters should be tuned (see also [4]).

CGHseg [9] requires the user to specify the threshold for choosing the appropriate penalty. The default value of -0.05 seemed appropriate for the Snijders data set (also shown in Figure 1 of the author's paper [9]). However, this value was clearly inadequate for the simulated data as it lead to detecting no breakpoints in essentially all data sets. Therefore, using the first 10 data sets, and the information on the true states, we choose the "best" threshold (-0.0025). Note that this can provide CGHseg a slightly unfair advantage over other methods (but see also a similar tuning of ACE), as some parameters are chosen so as to improve the performance using a small subset of the data. The output from the comparisons used to choose the threshold is in file "piccards.S.and.merging.selection.txt".

RJaCGH was run with six parallel chains, each with 60000 iterations of which the first 50000 were discarded as burn-in. For each run, two full chains were discarded by trimming (i.e., eliminating the two most extreme observations, one on each tail, with respect to the average estimated number of states of each chain). The parameters of the distributions of the candidates were selected automatically by a heuristic

Table 1: Confusion matrix

		Predicted		
		Gained	*No change*	*Lost*
**True**	*Gained*	TG	G_{nc}	G_l
**State**	*No change*	NC_g	TNC	NC_l
	Lost	L_g	L_{nc}	TL

approach that, within model, leads to an acceptance probability near 0.23 [10]. The parameters of the jumps between models were taken as the mean of the within model parameters.

1.3 Mapping of methods' output to gain/loss/no-change

Only ACE provides, directly, output labels that correspond to "gain/loss/no change" status of the genes. For DNAcopy, and as in [3], we post-processed the merge levels output, so the level with mean closest to zero, which is also the level with the largest number of observations, was assigned to the "no change" class (which is consistent with all assumptions in the normalization step, and most in the analysis step, that most genes/clones are not affected by copy number changes). The remaining levels were assigned to either "gain" or "lost" depending on whether the smoothed value was larger or smaller, respectively, than the "no change" class. Similar procedure was followed with HMM and BIOHMM after these methods returned their output.

CGHseg does not incorporate a mechanism for mapping segmented data into regions of loss/gain/no-change. We thus tried two approaches, one using the mergeLevels algorithm [3] as above, and another using a naive assignment of the most common class to the unaltered state, all segments with larger smoothed mean to the gained class, and all other to the loss class. The performance of these two approaches was compared using the first 10 data sets, with mergeLevels being clearly the best approach. The output from the comparisons used to choose the threshold and mergeLevels is in file "piccards.S.and.merging.selection.txt".

For RJaCGH, our method includes a some what similar approach. We consider as "no change" all states whose IQR (interquartile range) includes 0. After this step, we add the groups with posterior mean closes to 0 to the "no change" class until the proportion of observations in the no change class is no less than a pre-specified level (by default 0.65). This procedure is consistent with the assumptions in the normalization step that most genes/clones are not affected by copy number changes.

1.4 Statistics used to evaluate performance

We have evaluated performance of each method using four different statistics. To understand the statistics, it is useful to refer to table 1.

Correct classification rate The percentage of genes that are assigned to the right class. In table 1, the sum of all diagonal terms divided by the total number of clones. This is an overall estimate of how well a method is doing. This is likely to be the most relevant measure in every day usage, as it combines the measures below (and incorporates, for instance, trade-offs between False Discovery Rate and Sensitivity).

False Discovery Rate We define it in here as the number or mistakes made when we call something a gain or a loss: the number of no-changes among the clones Predicted to be gains or losses. In the table above,

$FDR = \frac{NC_g + NC_l}{TG + NC_g + L_g + G_l + NC_l + TL}$

(i.e., the sum of NC_g and NC_l divided by the total number of those predicted to be "gained" or "lost"). (Note that, in our comparisons, there was not a single case, for any method, were a true gain was predicted to be a lost, or vice-versa).

Specificity The probability of predicting no change when the true state is no change. In terms of table 1:

$Specificity = \frac{TNC}{NC_g + TNC + NC_l}$

Sensitivity The probability of predicting a gain (loss) outcome when the true state is gained (lost). Here we sum over both possible deviations from no change:

$Sensitivity = \frac{TG + TL}{TG + G_{nc} + G_l + L_g + L_{nc} + TL}$

It should be noted that there are ways to achieve, e.g., great False Discovery Rate, without being a good overall performer. For instance, by requiring very strong evidence to call something a loss, we can reduce the False Discovery Rate, at the expense of not identifying many changes as such (i.e., at the expense of lowering the sensitivity). Similarly, if a method predicts no change most of the time, the Specificity will be high at the expense of a low sensitivity.

2 Simulations

2.1 Simulation settings

We have used the same simulated data sets as Willenbrock and Fridlyand [3] used in their recent comparison of methods of aCGH analysis [3]. Details of the data are provided in the original paper [3]; briefly, these are data "(...) simulated to emulate the complexity of real tumor profiles" and designed to become "(...) a standard for systematic comparisons of computational segmentation approaches" [3, p. 4]. The authors simulated five hundred data sets based on the profiles of real tumor samples, and a sample-specific variance (between 0.1 and 0.2) was added to each sample. It is unlikely that these data were simulated under a model that is specifically well suited for our method. Other simulated data sets (or simulation approaches) did not seem

appropriate to compare alternative approaches; most papers that present simulated data do simulate the data under models that are the same (or very similar to) the model used to analyze the data. The simulations in [1] are useful for examining breakpoint detection, but not for questions related to the recovery of the correct "gained, lost, no change" label, and the simulations in [4] are too simplistic in their settings (only a single type of alteration added) and the number of points generated is too short (100). The 500 data sets of Willenbrock and Fridlyand [3], however, are suitable for examining recovery of true labels, are simulated based on real profiles to which varying levels of noise are added, and provide a sufficiently large and diverse data set to gain valuable information about the relative performance of different methods.

We downloaded the data [3] from `http://www.cbs.dtu.dk/~hanni/aCGH/`, and the actual file used was
`http://www.cbs.dtu.dk/~hanni/aCGH/20chromosome.simulated.data.RData`.
Each of the 500 simulations consisted of 20 chromosomes, with 100 clones in each chromosome. One hundred clones per chromosome are too few points (at least for most aCGH data for human samples) and make it hard to assess the effect of differences in spacing between clones. Thus, instead of using the 2000 clones as if divided in 20 chromosomes, we just regarded all the 2000 clones as if they came from the very same single chromosome which allows us to introduce fairly large numbers of missing data (i.e., variability in spacing).

None of the data sets above included variability in inter-gene distances which, as we argue in the paper, is an important feature of many real aCGH data sets, and a specific problem we try to address with our method. Therefore, to assess if our method does perform reasonably under varying inter-gene distance (and how it performs compared to other methods) we need to add inter-gene distance to the data set. Instead of modifying the original simulation models of [3], we have instead introduced "holes" (or missings) in the data thus replicating a situation where the data are generated according to the models in [3], but the actual observed data is a sample from the generated data (such as is the case with many aCGH platforms that show unequal coverage of different parts of the genome).

The "holes" or missing fragments in the data have been created with a very simple model: we choose at random 100 locations in the genome, and eliminate a contiguous segment of clones. The length of this segment is modeled with a Poisson distribution (so the actual length of the segment that is missing is drawn, randomly, from a Poisson distribution with parameter λ). This λ parameter determines the average number of missing points; in addition, as this is a Poisson distribution (where the variance is $= \lambda$), increasing λ results in an increase in the variance of the length of the missing fragments. We have used, for the λ parameter, the values 2, 5, 10, or 13. Thus, for each original data set, we obtain another four data sets, with a different number of missing data points. On average, the derived data sets have 10%, 25%, 50% and 65%. In other words, from the 500 data sets, we generate another 2000 data sets. Thus, of the 2500 data sets, each subset of 500 has an average number of missing points of 0% (in this case, 0 is not an average, but the actual number),

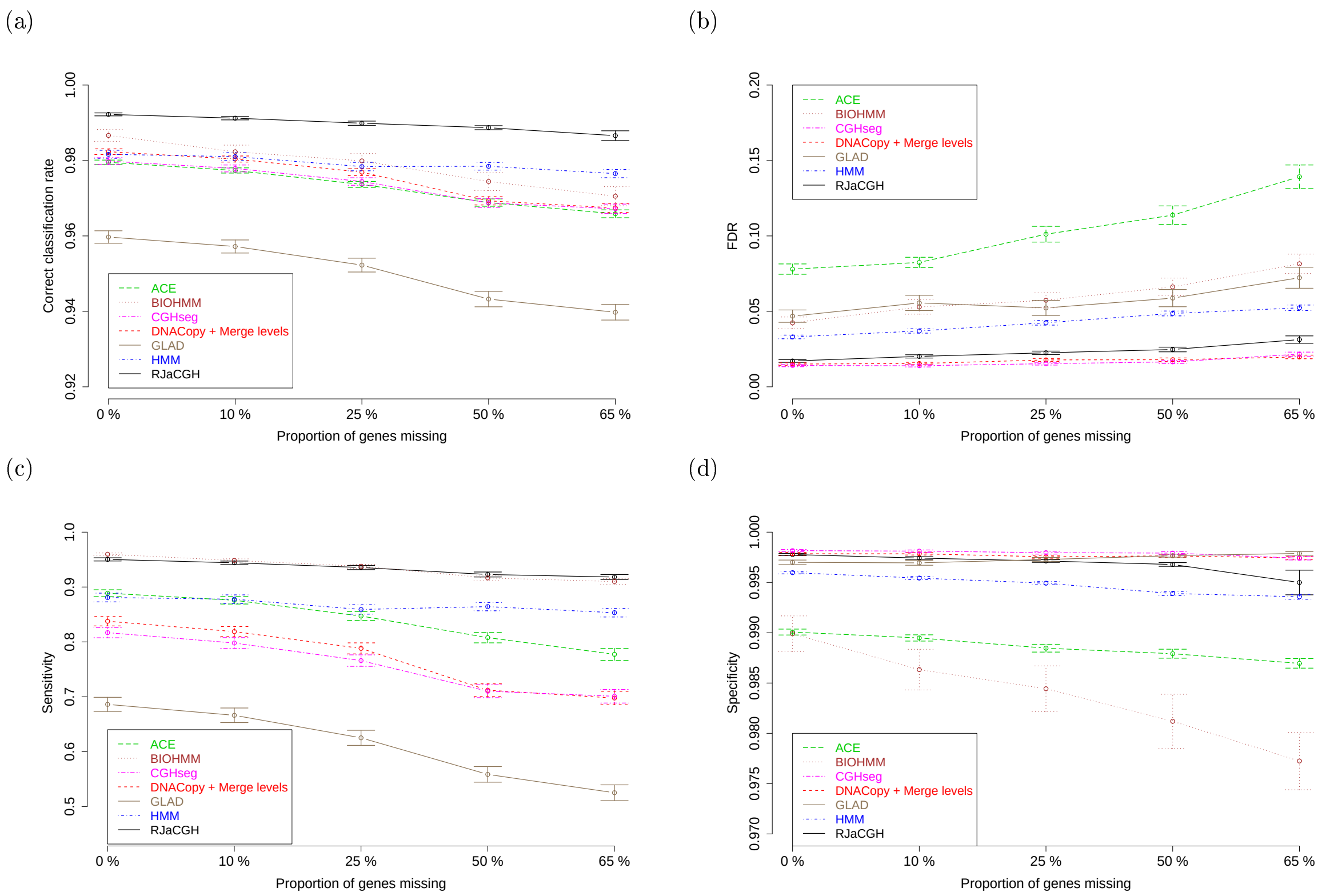

Figure 1: Comparative performance on the simulated data from [3] (see text for details). Relationship between the average value of the statistic and the variability in inter-gene distance (increases in the percentage of genes missing are directly related to increases in the variability in inter-gene distance). Shown are the mean and 95% confidence interval around the mean (based on 500 data sets). In panels (a), (c), (d), higher is better; in panel (b) lower is better.

10%, 25%, 50% and 65%. To minimize the variability in methods' comparisons, the derived data sets analyzed by all methods were the same.

2.2 Results and discussion

Results are shown in Figures 1, 2, 3.

Overall performance: Correct Classification Rate RJaCGH is better than any of the alternative approaches:

- The difference in performance between RJaCGH and alternative approaches increases as the variability in spacing between clones increases (i.e., as the proportion of missing genes increases). These patterns are seen in Figure 1 (a).
- The difference between RJaCGH and alternative approaches, is accentuated in Figures 2 and Figure 3: contrary to other methods, RJaCGH

does not suffer the same decrease in performance as the noise in the data increases.

False Discovery Rate The best performers are DNAcopy and CGHseg, and RJaCGH is the next best; all other methods suffer from much greater False Discovery Rates (Figure 1, (b)). As the noise in the data increases, however, the difference between RJaCGH and DNAcopy and CGHseg becomes smaller with RJaCGH being the method with smallest FDR at the highest noise levels (Figure 3 (b)). For all practical usages, however, differences between RJaCGH and DNAcopy and CGHseg in terms of FDR are probably negligible.

Note, however, that the good performance of DNAcopy and CGHseg with respect to False Discovery Rate is at the expense of a reduced Sensitivity (see next).

Sensitivity The largest sensitivity is achieved by BIOHMM at small values of noise in the data and by RJaCGH with higher noise levels (see panel (c) in all Figures). Over all levels of noise in the data, however, the performance between RJaCGH and BIOHMM (Figure 1 (c)) is indistinguishable, but clearly superior to other methods. The good performance of BIOHMM with respect to Sensitivity, however, is achieved at the expense of its high False Discovery Rate and low Specificity (see below).

Specificity As could be expected from the definition of Specificity and False Discovery Rate, the patterns of Specificity are similar to those commented above for False Discovery Rate.

In summary, RJaCGH has the largest correct classification. For some specific statistics, RJaCGH can be second (but very close) to some approaches; these other approaches, however, perform poorly in the other performance statistics. Overall, therefore, RJaCGH is the best performing method when considering the four available statistics.

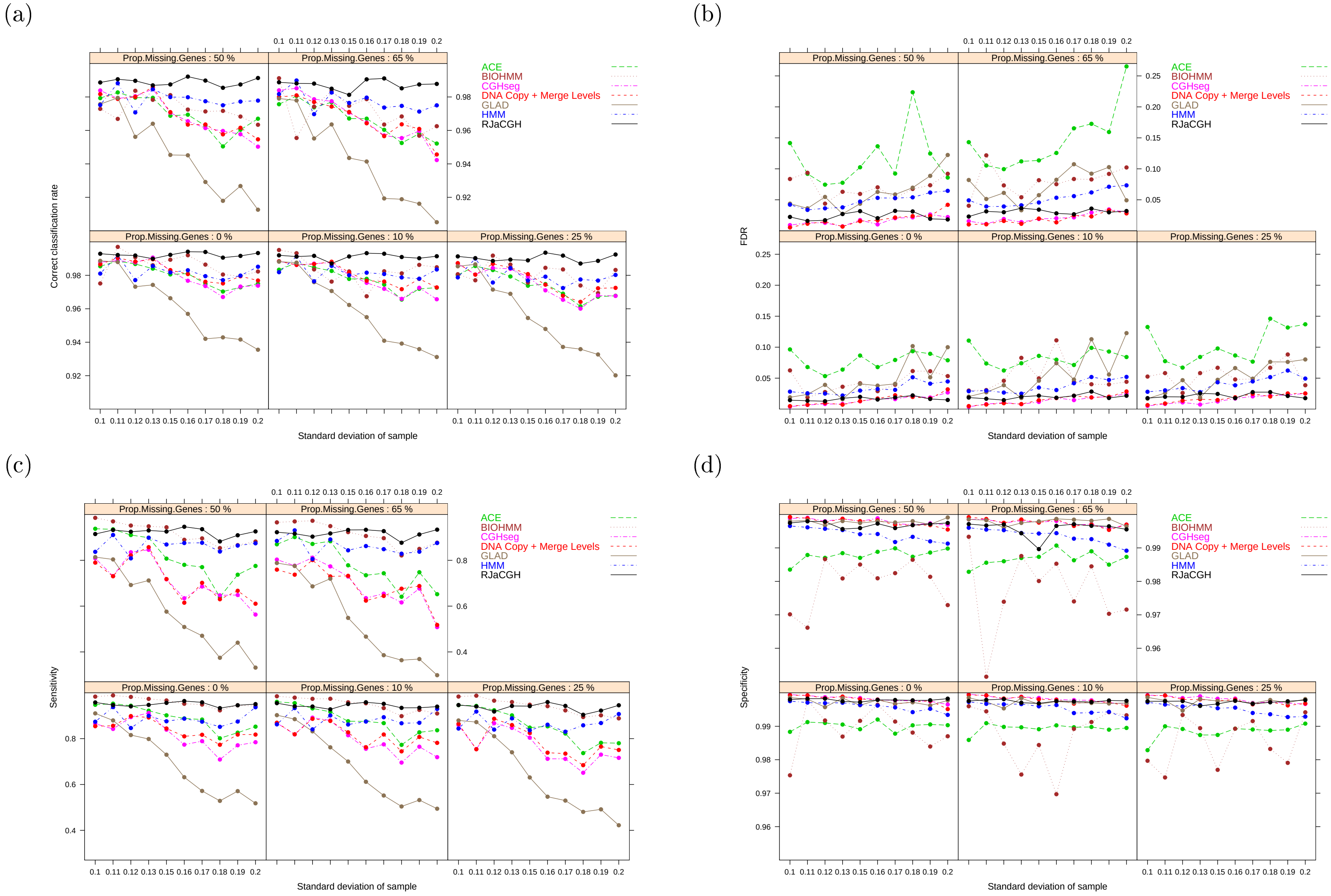

Figure 2: Analysis of simulated data: conditioning on variability of inter-gene distance. Analysis of data from Willenbrock and Fridlyand [3] (see text for details on addition of gaps). For each level of average number of missing genes (0, 10, 25, 50, 65 %) or, equivalently, for increasing levels of variance in the distance between clones, we compute the mean of the statistic at ten equally spaced levels of noise in the data (i.e., the 500 data sets have been divided in 10 groups according to their noise, so that the midpoints of each interval are $0.105, 0.115, 0.125, \ldots, 0.185, 0.195$). Therefore, each point in the figure corresponds to the mean from about 50 samples.

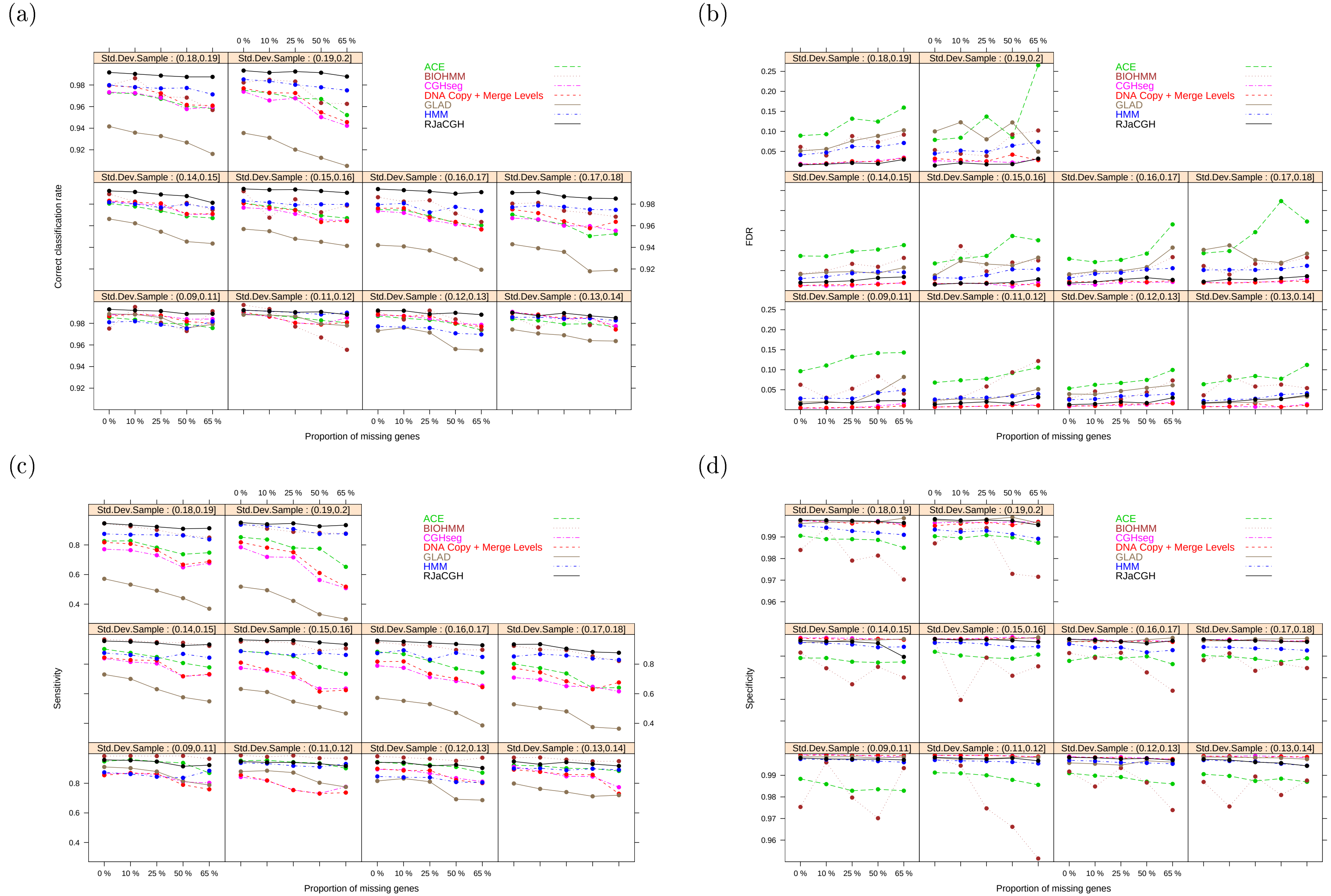

Figure 3: Analysis of simulated data: conditioning on sample noise. Analysis of data from Willenbrock and Fridlyand [3] (see text and Figure 2 for details). The noise (standard deviation) of each sample is split into ten non-overlapping ranges, and each panel shows the average value of the statistic vs. the proportion of missing genes (i.e., increasing levels of variance in inter-gene distance) for a given sample noise.

3 Real data from Snijders et al.

We have also analyzed the well known nine cell lines from Snijders et al. [11] available from
http://www.nature.com/ng/journal/v29/n3/suppinfo/ng754_S1.html and we have compared the results from our method with the known ploidy, as provided by Snijders et al.

Figure 4 shows the comparative performance of each of the methods. From the figure we see that RJaCGH has performance comparable to that of the best method for each statistic.

As an example of the type of output provided by RJaCGH, Figure 5 shows the results of one analysis for the complete genome of the cell line gm03563. Panel a) indicates a large posterior probability of a model with four hidden states; two of the

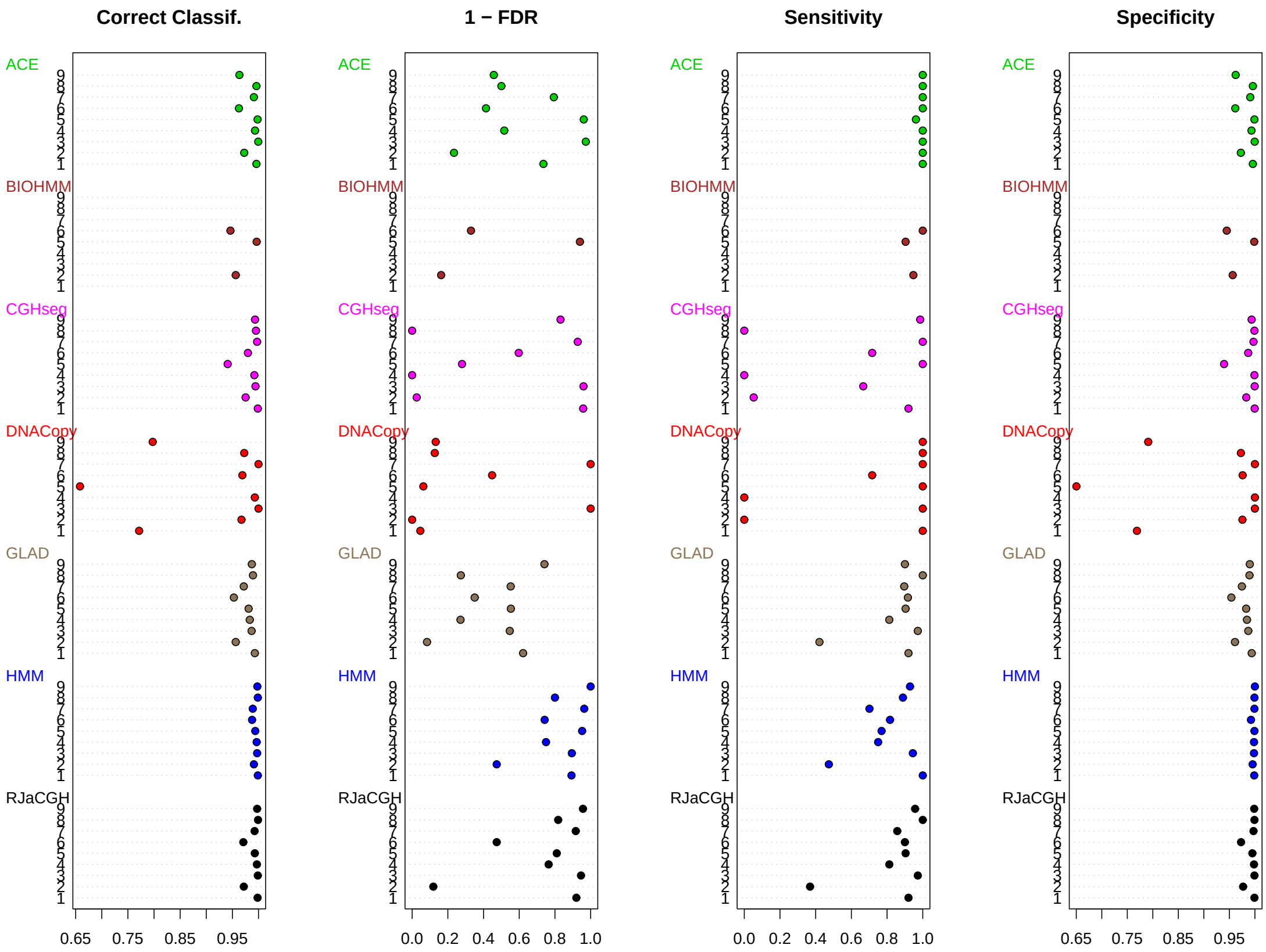

Figure 4: Comparative performance on the nine cell lines from Snijders et al. [11]. We show the value of the performance statistics for each cell line (numbered 1 to 9, which correspond to gm01524, gm01535, gm01750, gm03134, gm03563, gm05296, gm07081, gm13031, gm13330, respectively). In all these figures, "larger is better" (note we use 1- FDR, not FDR). Only three values are shown for BIOHMM, as the rest of data lead to crashes in the program.

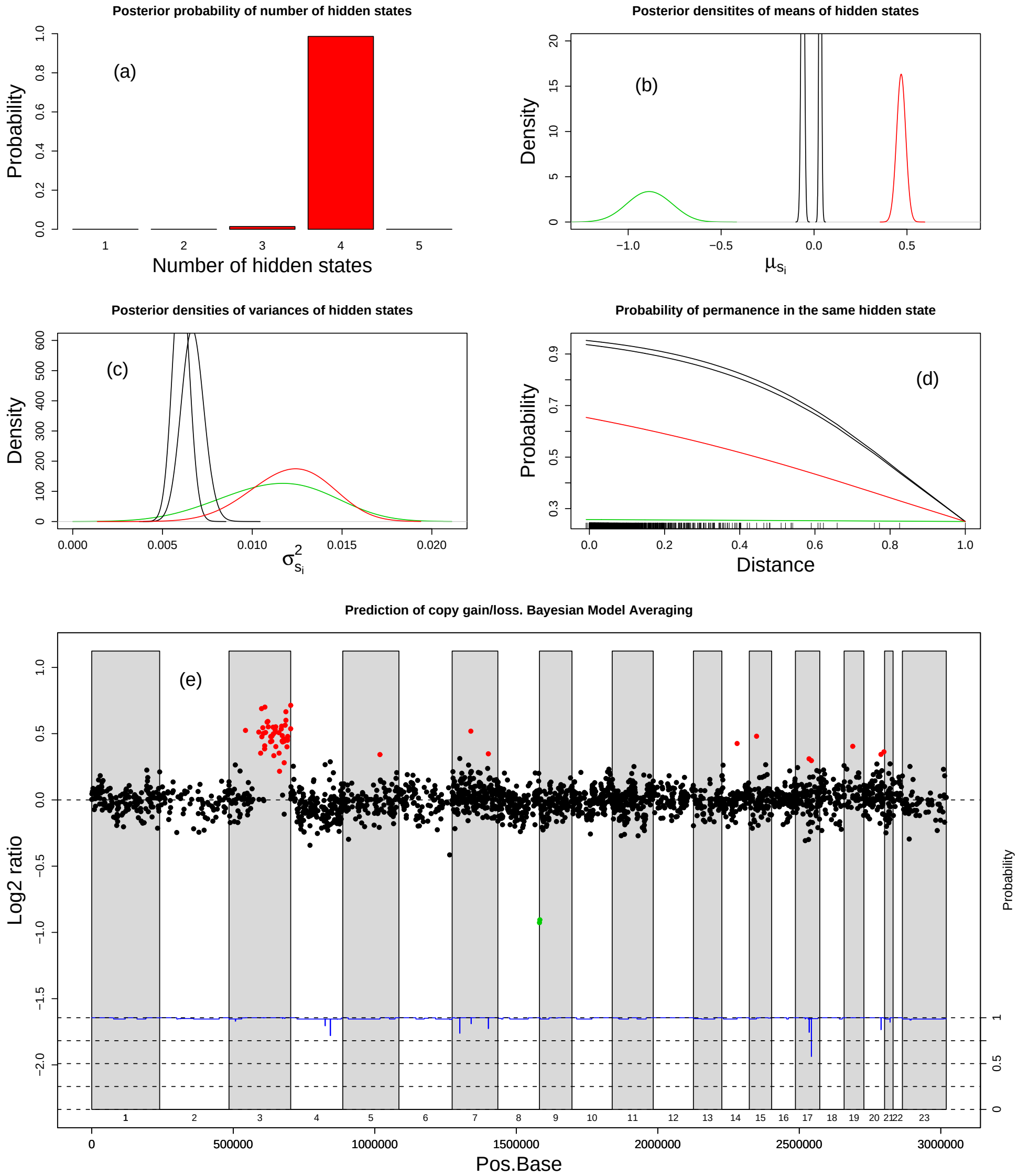

Figure 5: Results of the RJaCGH analysis of gm03563 cell line from Snijders. Results shown are from four parallel chains; see text for details about other parameters. The lower panel shows the results from the Bayesian Model Averaging step (see text); black dots correspond to genes classified as 'normal' or non-changed, red dots to genes classified as 'gained' and green dots to genes classified as 'losses'; the lower blue line shows the posterior probability for every gene of belonging to the predicted state. The vertical alternating white and grey bars denote the different chromosomes with the chromosome number shown at bottom.

states of the four-state model, however, are extremely close to each other (panel b) and, because of their posterior means (panel b) and variances (panel c) we consider them to represent the same biological state of no change in copy number. The other two states are well separated, with posterior means clearly negative or positive, so

we regard them as biological states of loss and gain of copy number. Note that the component that represents the hidden state of loss is assigned to only two genes (panel e, green dots), exactly the same two genes whose true state is loss [11]. Panel d) shows that the probability of remaining on the same state decreases as distance increases, eventually becoming $0.25 (= 1/\text{Number hidden states})$. Finally, panel e) shows the results from the Bayesian Model Averaging. This is a particularly clear-cut model, as the posterior probabilities that each gene belongs to the state with highest posterior is very high (the lower blue line is > 0.9 for almost all genes).

4 Implementation and analysis

We have implemented RJaCGH using C (for the sweep algorithm) and R [12]. The code is available from CRAN
(`http://cran.r-project.org/src/contrib/Descriptions/RJaCGH.html`)
and from the Asterias site (`http://www.asterias.info`). All analysis and comparisons have been done in R; we have used the BioConductor (`http://www.bioconductor.org`) packages DNAcopy (for the DNAcopy method) by E. S. Venkatraman and Adam Olshen, aCGH (for the HMM method and mergeLevels algorithm) by Jane Fridlyand and Peter Dimitorv, snapCGH (for BioHMM) by Mike L. Smith, John C. Marioni, Steven McKinney and Natalie P. Thorne, GLAD by O. Huppe, and tilingArray (for CGHseg with additional modifications to use the original penalization) by W. Huber; we have also used a version of ACE implemented by O.M.R. in R and C.

5 Additional files: input, output, and code

All the code for the analysis, simulations, and figure preparation, as well as all input and output files for each method and every run are available from
`http://asterias.bioinfo.cnio.es/RJHMM_20061110_1437_smf/AdditionalFiles.html`.

References

[1] Olshen AB, Venkatraman ES, Lucito R, Wigler M: **Circular binary segmentation for the analysis of array-based dna copy number data.** *Biostatistics* 2004, 5:557–572.

[2] Lingjaerde OC, Baumbusch LO, LiestÃ¸l K, Glad IK, Borresen-Dale AL: **Cgh-explorer: a program for analysis of array-cgh data.** *Bioinformatics* 2005, 21:821–822.

[3] Willenbrock H, Fridlyand J: **A comparison study: applying segmentation to array cgh data for downstream analyses.** *Bioinformatics* 2005, 21:4084–4091.

[4] Lai WRR, Johnson MDD, Kucherlapati R, Park PJJ: **Comparative analysis of algorithms for identifying amplifications and deletions in array cgh data.** *Bioinformatics* 2005, 21:3763–3770.

[5] Fridlyand J, Snijders AM, Pinkel D, Albertson DGa: **Hidden markov models approach to the analysis of array cgh data**. *Journal of Multivariate Analysis* 2004, 90:132–153.

[6] Marioni JC, Thorne NP, Tavaré S: **Biohmm: a heterogeneous hidden markov model for segmenting array cgh data.** *Bioinformatics* 2006, 22:1144–1146.

[7] Daruwala RS, Rudra A, Ostrer H, Lucito R, Wigler M, Mishra B: **A versatile statistical analysis algorithm to detect genome copy number variation.** *Proc Natl Acad Sci U S A* 2004, 101:16292–16297.

[8] Hupé P, Stransky N, Thiery JP, Radvanyi F, Barillot E: **Analysis of array cgh data: from signal ratio to gain and loss of dna regions.** *Bioinformatics* 2004, 20:3413–3422.

[9] Picard F, Robin S, Lavielle M, Vaisse C, Daudin JJ: **A statistical approach for array cgh data analysis.** *BMC Bioinformatics* 2005, 6:27.

[10] Gelman A, Carlin JB, Stern HS, Rubin DB: *Bayesian Data Analysis, Second Edition*. Chapman & Hall/CRC, 2003.

[11] Snijders AM, Nowak N, Segraves R, Blackwood S, Brown N, Conroy J, Hamilton G, Hindle AK, Huey B, Kimura K, Law S, Myambo K, Palmer J, Ylstra B, Yue JP, Gray JW, Jain AN, Pinkel D, Albertson DG: **Assembly of microarrays for genome-wide measurement of dna copy number**. *Nat Genet* 2001, 29:263–264.

[12] R Development Core Team: *R: A Language and Environment for Statistical Computing*. R Foundation for Statistical Computing, Vienna, Austria, 2006.
